# Future groundwater potential mapping using machine learning algorithms and climate change scenarios in Bangladesh

**DOI:** 10.1038/s41598-024-60560-2

**Published:** 2024-05-06

**Authors:** Showmitra Kumar Sarkar, Rhyme Rubayet Rudra, Swapan Talukdar, Palash Chandra Das, Md. Sadmin Nur, Edris Alam, Md Kamrul Islam, Abu Reza Md. Towfiqul Islam

**Affiliations:** 1 Department of Urban and Regional Planning, Khulna University of Engineering & Technology (KUET), Khulna, 9203 Bangladesh; 2 Department of Geography, Asutosh College, University of Calcutta, Kolkata, 700026 India; 3 Department of Geography, Texas A&M University, College Station, USA; 4 Faculty of Resilience, Rabdan Academy, 22401 Abu Dhabi, United Arab Emirates; 5 Department of Geography and Environmental Studies, University of Chittagong, Chittagong, 4331 Bangladesh; 6 Department of Civil and Environmental Engineering, College of Engineering, King Faisal University, AlAhsa, 31982 Saudi Arabia; 7 Department of Disaster Management, Begum Rokeya University, Rangpur, 5400 Bangladesh; 8 Department of Development Studies, Daffodil International University, Dhaka, 1216 Bangladesh

**Keywords:** Groundwater potentiality, Data mining, Ensemble machine learning, Remote sensing, Climate change, Climate sciences, Environmental sciences, Hydrology

## Abstract

The aim of the study was to estimate future groundwater potential zones based on machine learning algorithms and climate change scenarios. Fourteen parameters (i.e., curvature, drainage density, slope, roughness, rainfall, temperature, relative humidity, lineament density, land use and land cover, general soil types, geology, geomorphology, topographic position index (TPI), topographic wetness index (TWI)) were used in developing machine learning algorithms. Three machine learning algorithms (i.e., artificial neural network (ANN), logistic model tree (LMT), and logistic regression (LR)) were applied to identify groundwater potential zones. The best-fit model was selected based on the ROC curve. Representative concentration pathways (RCP) of 2.5, 4.5, 6.0, and 8.5 climate scenarios of precipitation were used for modeling future climate change. Finally, future groundwater potential zones were identified for 2025, 2030, 2035, and 2040 based on the best machine learning model and future RCP models. According to findings, ANN shows better accuracy than the other two models (AUC: 0.875). The ANN model predicted that 23.10 percent of the land was in very high groundwater potential zones, whereas 33.50 percent was in extremely high groundwater potential zones. The study forecasts precipitation values under different climate change scenarios (RCP2.6, RCP4.5, RCP6, and RCP8.5) for 2025, 2030, 2035, and 2040 using an ANN model and shows spatial distribution maps for each scenario. Finally, sixteen scenarios were generated for future groundwater potential zones. Government officials may utilize the study’s results to inform evidence-based choices on water management and planning at the national level.

## Introduction

Water scarcity is increasing throughout the world due to the effects of increasing groundwater abstraction and climatic change. One of the most important resources on earth, groundwater accounts for around 34 percent of the world’s freshwater supply. It is the primary water source and is regarded as less contaminated than other sources. A few years ago, groundwater was considered as a safe water supply. However, in recent years, the state of water has revealed that groundwater is extremely sensitive to inanition in many places around the world, particularly in developing countries^1^. All waters found below the surface of the ground are referred to as groundwater, or subterranean water^2^. In addition to being essential for human survival, groundwater also serves several practical functions in areas including agriculture, industry, and daily home usage. Rain or snowmelt that percolates through the soil or the pore spaces of surrounding rocks replenishes groundwater in a natural way. Groundwater is one of among the most crucial components of water supplies for human society^3^. 26 percent of the world’s renewable freshwater supplies come from groundwater. It provides water for homes, commercial, industrial, agricultural, and other development projects^4^. The global effects of climate and weather change, prolonged drought conditions, and an absence of precipitation have all contributed to an alarming rise in demand for groundwater. The groundwater table quickly dropped due to excessive consumption, and the resource’s sustainability was quickly depleted^5^. However, ineffective groundwater management has had negative consequences, including a decline in water quality, sinking of water levels, poorer crop yields, etc.^5,6^. Human activities have the capacity to bring pollutants into groundwater, which can have adverse effects on the water’s quality and render it unfit for consumption^7^. Several nations reliant on groundwater as a sustainable resource are apprehensive about the diminishing quality and quantity of water in the aquifer^8^. Due to the economic and technological limitations in emerging economies, groundwater is frequently utilized without adequate treatment. Such activity poses a significant threat to human health because of exposure to fluoride, nitrates, and various other contaminants found in groundwater^9^. Water resource modelling has become a powerful tool for management that is essential for comprehending hydrological data, describing groundwater systems, and forecasting how they will react to pollution and external stresses^10^.

Groundwater potential zone modeling is critical for sustainable water resource management since it helps to allocate this key resource more efficiently for agriculture, industry, and home usage. It permits the identification of locations appropriate for groundwater extraction while preserving the resource, guaranteeing long-term growth and resilience in the face of droughts or climate change^6,11,12^. Traditional approaches for locating areas with high groundwater potential zone modeling rely heavily on expensive and time-consuming ground surveys^6^. GIS and Remote sensing is a very useful tool for modeling groundwater water potential zones. Researchers have employed various geospatial techniques for modeling groundwater potential zones such as : the AHP method^4,6^, the fuzzy logic^13^, combination of GIS and fuzzy logic^14^, hybrid multi-criteria approach in Google Earth Engine^15^, AHP and Fuzzy logic based technique^16^ etc. Nowadays, the use of machine learning algorithms has increased a lot in the field of groundwater potential zone modeling. Machine learning (ML) algorithms outperform traditional approaches in groundwater potential zone modelling because of their ability to manage complex, non-linear interactions across varied datasets and adapt to changing hydrogeological circumstances. Unlike previous techniques, ML algorithms easily deal with non-linearity, making them crucial for mapping groundwater potential zones and improving decision-making in sustainable water resource management^17–19^. The most appropriate ML algorithm is determined by dataset features, and comparison studies are critical for identifying the method that works best in a certain environment. Various machine learning algorithms such as the: artificial neural network (ANN) algorithm^20,21^; function model^22^; the decision tree^23^; the greatest entropy and random forest (RF) models^24^; and shannon entropy (SE) to Geographic Information Systems (GIS)^25^, decision tree^18^, random forest (RF)^26^, deep learning^27^, support vector machine learning model (SVM)^17^ etc. have been used over the years to detect the groundwater potential zones. ML techniques such as RF, and ANN excel in automating the modelling process, capturing complicated patterns, and making accurate predictions^19,26,27^. They provide a data-driven strategy that identifies feature relevance for a better understanding of groundwater impacting elements. Due to its accuracy and low cost perspectives the use of ML alongside geospatial tools have become popular over the years in many developing countries especially in the South Asian countries. Ensemble Modelling Framework for groundwater level 2 prediction in Bihar^19^, groundwater Arsenic and health risk prediction model using ML in Pakistan^28^, mapping of groundwater productivity potential with ML algorithms in the provincial capital of Baluchistan, Pakistan^29^, water quality analysis with the help of ML algorithms in Sri Lanka by^30^ shows the growing interest of ML algorithms in these countries to detect the groundwater modeling. Prediction of groundwater level changes has been done in several circumstances^31,32^,. Furthermore, researchers predicted how various climate change scenarios might affect groundwater levels^33^.

Bangladesh is one of the most over populated^34–36^, polluted^37–39^ and disaster-prone countries in the world^40–43^. Due to its riverine geography and tropical climate, Bangladesh is fortunate to have access to a considerably greater variety of water sources. Despite issues with arsenic, iron, manganese, and microbial pollution, groundwater is believed to be safer to drink than surface water^44–46^. Groundwater is a major source of drinking water and irrigation for the people of Bangladesh. Groundwater is becoming a significant issue in Bangladesh. Day by day, groundwater is polluted by pathogens and agrochemicals. Furthermore, the groundwater in coastal locations is unsuitable for irrigation and drinking due to rising sea levels and saline soils. The nation experiences a severe water shortage throughout the year, particularly during the dry season, as a result of excessive groundwater extraction, unmanaged surface water contamination, the effects of disasters brought on by climate change, saline intrusion, etc.^6,47–49^. Additionally, the amount of agricultural land is constantly declining, and the increase in impermeable surfaces caused by vegetation has only made matters worse^50–52^. A sizeable population is hence regularly exposed to the risks caused by water shortages and poor water quality. Therefore, it is essential to recognize potential groundwater sources and manage them effectively^6,53^. Groundwater mapping and zoning are important for sustainable water resource management. Mapping and zoning are essential in Bangladesh, where groundwater is vital to agriculture, drinking water, and industry. The approach helps discover high-potential groundwater sites for efficient and sustainable agriculture while limiting overexploitation in sensitive locations. Mapping helps manage water supplies in highly populated regions to fulfil household needs^54^. Mapping and zoning are also necessary for informed decision-making due to climatic vulnerabilities, salt intrusion problems, and groundwater quality difficulties. Authorities may promote sustainable water usage, resilient urban development, and groundwater protection by identifying high-potential areas. Groundwater mapping and zoning strengthen communities, ecosystems, and enterprises that use groundwater^11^.

Several studies have been conducted in different regions of Bangladesh to identify the groundwater potential zone, including a comparison of the accuracy of the FR and SE model a AHP, and a GIS^11,12,55^, among others. There has been an increase in the use of machine learning algorithms to identify potential groundwater zones in recent years^11,21,24,25,56,57^. Conversely, climate change is a major problem in Bangladesh^58^. However, there has been a dearth of studies comparing the efficacy of different machine learning models for pinpointing groundwater potential zones and the impact that future climate change (RCP2.5, RCP4.5, RCP6, and RCP8.5) may have on groundwater levels in Bangladesh. The ability of machine learning algorithms to predict future precipitation under climate change scenarios, thus, has a considerable impact^33^. With this research, we want to fill in the blanks. The study’s primary objectives are: a) locating areas with high potential for groundwater use three distinct machine learning models ANN, logistic regression (LR), and a logistic model tree (LMT), all of which are based on a total of fourteen variables; b) predicting potential changes in groundwater levels in the study region in response to numerous climate change scenarios (RCP2.6, RCP4.5, RCP6, and RCP8.5); and c) comparing three machine learning algorithms using the ROC curve to determine which model is the best match for our study area. The results of this study will be used to identify the regions of Bangladesh that are suffering groundwater shortage and to choose the most appropriate model for mapping groundwater potential zones. Inspiring them to use the required model to locate likely groundwater zones, it will aid scientists in understanding the possible consequences of climate change on groundwater levels and give important data for Bangladesh’s decision-makers and managers.

## Methods and materials

### Study area

**Figure 1:**
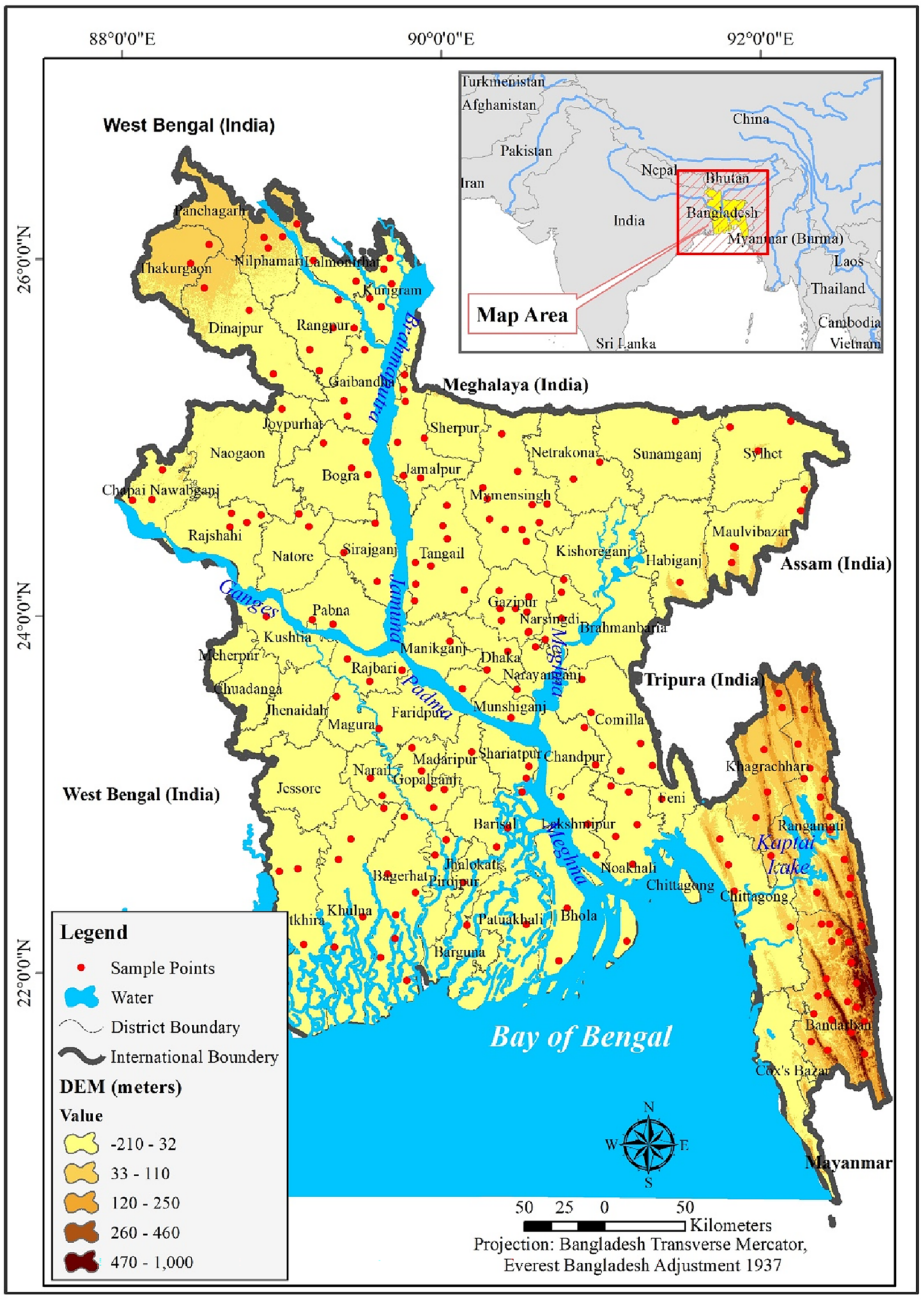
Study area; prepared by the authors using ArcGIS software version 10.5, (https://www.esri.com/en-us/arcgis/products).

Bangladesh, a country situated in Southeast Asia (Fig. 1), is divided into seven distinct climatic sub-regions based on its geography and weather patterns. The country experiences seasonal changes due to its humid subtropical monsoon climate, with extreme weather swings being a typical feature. The average daily long-term values for relative humidity, lowest temperature, maximum temperature, wind speed, net radiation, and evapotranspiration are 80%, 21.39 °C, 29.94 °C, 1.32 ms-1, 10.44 MJm-2 day-1, and 3.72 mmd-1, accordingly. Western Bangladesh is drier than other regions. Agriculture in Bangladesh heavily relies on groundwater irrigation, primarily for growing staples such as Boro rice during the dry season. Shallow tubewells are the main source of groundwater for irrigation, and there are presently more than 1.5 million of them worldwide, up from 100,000 in the early 1980s. However, concerns regarding unsustainable groundwater consumption have been raised due to declining levels of groundwater, particularly in the Barind region of northwest Bangladesh. Experts suggest that continuing to irrigate the Barind region using shallow aquifers is unsustainable, as the recharge is inadequate in some areas. While groundwater extraction in Dhaka is likewise problematic for urban and industrial applications, it accounts for only a small portion of the country’s total water consumption compared to irrigation. Excessive groundwater use has been linked to falling water tables in northeastern Bangladesh, but there is little evidence that the use of groundwater is unsustainable over much of Bangladesh. According to recent research, the northwest area does not have a groundwater shortage, and present water use is only approximately two-thirds of the safe output. While there are concerns regarding unsustainable groundwater consumption in certain regions, it is vital to develop sustainable water management policies and practices that balance the needs of agriculture, industry, and urban areas to ensure the continued availability of this critical resource in Bangladesh.

### Materials and groundwater potentiality inventory

Fourteen parameters were used in the study (i.e., Curvature, drainage density, slope, roughness, rainfall, temperature, relative humidity, lineament density, land use and land cover, general soil types, geology, geomorphology, topographic position index (TPI), Topographic Wetness Index (TWI)). The US Geological Survey provided topographic and land use/land cover data, the Bangladesh Meteorological Department provided weather data, and the Geology Survey of Bangladesh provided geology and soil data, the sample points of field survey were taken from Bangladesh Water Development Board.

Several scientists have mapped groundwater potential by cataloging the locations of springs, wells, and quant. In this analysis, groundwater potential was accounted for as well locations. 200 points representing wells were gathered from different sources and a thorough site survey to create the inventory graph for the research area. As a first stage, we collect information that is not groundwater but is similar to the information utilized in the potential groundwater model. The decision was based on the field survey and the same amount of weight was given to the non-groundwater data (200). All data, groundwater and otherwise, has been arbitrarily split into an 80:20 calibrating and test dataset split. Models are calibrated and validated using both groundwater and non-groundwater training data and testing data, respectively. The data set were divided into two parts with the binary number 0 and 1. Binary number 0 were used as non-groundwater data and 1 was used as groundwater data. Total 160 sample points were used as the input data for training on the contrary, 40 sample points were used the input for the testing the dataset.

### Method for groundwater potentiality modeling

#### Artificial neural network

ANN builds its models on top of previously observed behavioral patterns. It has multiple layers of organization, including input, covered, and output, and processing units like neurons^59^. The biological neuron, together with its simplified characteristics, serves as the primary building block of artificial neural networks. These were created as a rudimentary mathematical model that mimics the functioning of the human brain^60^. Attachment weights connect neurons in one layer to those in the next. The data is transferred to the final layer (hidden layer) as the intermediate layer’s output. The input layer receives information, whereas the output layer generates the ANN model’s real outcomes. Lower layer data is received and transmitted to their corresponding nodes in higher levels. A weighted set of inputs is processed by hidden neurons to create an intermediate output. The hidden and output neurons’ outputs are derived using activation functions in the ANN model^61,62^. Bias values and the input weight matrix are used to determine the neuron’s output. The meat of an ANN modeling method consists of two steps: creating the network and adjusting the link weights. The literature research indicates that water engineering is only one of several fields where the backpropagation training method has found widespread use. The effectiveness of the ANN model is evaluated based on the precision of its predictions. Subsequently, the weights of the model are established, therefore minimizing the disparity between the observed and anticipated values. To minimize error values, the weights and biases are adjusted when the observed value deviates from the output. This study utilized meta-heuristic optimization strategies because to the slow convergence rate of the backpropagation method. The vector x is formed by n inputs, denoted as × 1, × 2, …, xn. The input is multiplied by the weight parameter, which may have a positive or negative value. The input neuron × 0, which represents the bias, is assigned a weight of × 0^63^. The sum of all weighted inputs yin indicates the internal potential of the neuron:

1 $$Yin={\sum }_{i=0}^{n}WiXi$$

Equation 1 shows the sum of all functions. The aggregated total is subsequently sent through a neuron activation function y = f (y in) to generate the final output of the neuron. This can act as a trigger for neurons in the subsequent layer of the neural network. Neurons create a neural network when they establish connections with each other. The connection mechanism operates in such a way that the output of one neuron serves as the input for another. The neurons in the network are organised into layers^64^. Each network has an input layer, an output layer, and any number of hidden layers. The ability to modify the weights between neurons is a key property of neural networks. The weights in the network are reinforced or weakened based on the correct or incorrect answers^63^. There are three main categories of learning algorithms: supervised, unsupervised, and reinforcement. Every neuron in a multilayer perceptron network functions similarly to a perceptron. A sigmoid function is the most popular activation function for neurons in multilayer perceptron, which are differentiable continuous functions^65,66^.

2 $$f(x)=\frac{1}{1+{e}^{x}}$$

Equation 2 shows the multilayer perceptron in neurons. Multilayer perceptron lead to the full interconnection of neurons—each neuron in the layer is connected to all the neurons of the above (following) layer. Fourteen parameters (i.e., curvature, drainage density, slope, roughness, rainfall, temperature, relative humidity, lineament density, land use and land cover, general soil types, geology, geomorphology, topographic position index (TPI), topographic wetness index (TWI)) were used as an input in neural network package.

#### Logistic regression

Comparing the probability of an event happening to a group of reasonable predictions is one of the most common applications of the likelihood ratio (LR) statistical method^67^. In the situation of groundwater potentiality prediction, where the occurrence has been classed as groundwater or non-groundwater, LR attempts to discover the most effective method for investigating the link between a certain group of conditioning variables and the presence or absence of groundwater^68,69^. Furthermore, LR has been considered to have excellent predictive performance in classification applications since it maximizes the likelihood function utilizing its convergence criteria^67,70^. In the LR model, the conditioning variables refer to the traits or attributes that are utilized for making predictions. The model takes into account these input factors while calculating the likelihood of a specific class. In normal logistic regression, relevant characteristics are chosen based on their impact on predicted accuracy and statistical significance^71^. The process of conditioning variable selection may employ approaches such as feature significance analysis, stepwise selection, or regularization methods, depending on the exact details of the implementation. 80% of the data from the fourteen parameters were selected as the training data, while the remaining data was chosen as the testing data. The testing data was then utilized to create the ROC curve.

3 $$P\left( {y = 1/X} \right) = 1 / \left( {1 + e^{{ - (\beta_{0} + \beta_{1} x_{1} + \beta_{2} x_{2} + ... + \beta nxn}} } \right)$$

In Eq. 3, P (y = 1/X) is the probability of belonging to class 1 given the input features. e is the base of the natural logarithm. *β*0, *β*1, *β*2, …, *βn* are the coefficients learned by the logistic regression model. × 1, × 2, …, *xn* are the input feature^72,73^.

#### Logistic model tree

A logistic tree model (LTM) is a kind of classification model that combines logistic regression (LR) with decision tree learning^74,75^. In the logistic variant, an LR model is constructed at each tree node using the LogitBoost technique^75^, the CART method is then used to prune the tree once it has been constructed. In order to avoid overfitting the training data, the LMT selects a set of LogitBoost iterations with the help of cross-validation. This keeps the model from seeming to be too accurate. LogitBoost uses least-squares additive logistic regression to make predictions for many classes at once. The LogitBoost algorithm is utilized to construct logistic regression models at each node of the tree. LogitBoost is a specialized boosting technique tailored for logistic regression issues. LogitBoost employs least-squares additive logistic regression inside the framework of the long-term memory (LTM) to generate predictions for many classes concurrently. It enhances the performance of logistic regression models by repeatedly adjusting them to the discrepancies of the previous iterations^75^. Boosting improves the predicted accuracy of LR models by assigning greater importance to incorrectly categorized examples at each iteration, hence rectifying mistakes and strengthening the model. The tree is built by utilizing LR models at each node, and then the CART approach is used for pruning. Pruning is essential for avoiding overfitting, and in the case of CART, it commonly use parameters like Gini impurity or information gain to determine which branches of the tree should be cut^50,51^. To mitigate overfitting, one can employ cross-validation to determine the optimal number of LogitBoost rounds. Through assessing the model’s efficacy on a validation set, the algorithm may ascertain the ideal amount of iterations that avoid the model from becoming excessively intricate and customized to the training data^77^. Here the 80% data from the fourteen parameters are chosen as the training data and rest of the data were chosen as the testing data which was later used for making the ROC curve. The Logistic Model Tree combines decision tree learning with logistic regression. The basic structure of an LMT involves decision nodes, which represent tests on input features, and logistic regression models at the leaves. LMT uses the Eq. 3 in their decision tree node.

### Validation of the models

On the ROC curve, the sensitivity (TPR) is shown along the y-axis, and the specificity (FPR) is shown along the x-axis for a variety of test data cut-off points. In most representations, it has the form of a square box with axes numbered 0–1. A diagnostic test’s inherent validity may be assessed using the AUC sensitivity and specificity statistic. AUC = 1 means the diagnostic test can distinguish groundwater from non-groundwater with 100% accuracy. Equal sensitivity and specificity are implied by the absence of false positives or negatives. This is really unlikely. As test performance increases, AUC becomes closer to one. The square is split into two portions of 0.5 square meters by the diagonal from (0, 0) to (1, 1). The likelihood that this line’s ROC can tell non-groundwater from groundwater is 50/50. Since an AUC value of 0 suggests that the test mistakenly identified all of the groundwater participants as negative and all of the non-groundwater participants as positive, the bare minimum value for the AUC should be 0.5. When the results of the test are flipped, the region that was previously equal to 0 becomes equal to 1, making an otherwise completely flawed test accurate. Model performance in this situation must be more than 70% AUC^57^.

### Future climate change models

Global warming has altered air and ocean currents. This changed global precipitation and temperature patterns. Thus, groundwater depletion increases with global warming^78^. A shift in intense rainfall events may increase flooding hazards in terrain unfavorable for groundwater recharging^78^.

Researchers tried stochastic connections. General circulation model’s (GCM) predict climates. The groundwater potential models projected future rainfall, temperature, LULC, and other fixed factors. This study utilized CCSM-4 data from the fourth IPCC Assessment Report (AR) to address climate change in research. This analysis used AR5 representative concentration pathways (RCP) 2.5, 4.5, 6.0, 8.5 climate scenarios. RCPs depict air GHG concentrations and paths to get there. Due to greenhouse effect warming, they will induce radiation forcing by 2100. RCP2.5, 4.5, 6.0, and 8.5 are four IPCC RCPs based on greenhouse gas concentrations.

The NCAR GIS Initiative Climate Change Scenario site (https://gisclimatechange.ucar.edu) provides free data for this research. This research examined groundwater potentiality using climatic factors like rainfall for 2025, 2030, 2035 and 2040 under RCP2.5, 4.5, 6.0 and 8.5.

### Future groundwater potential models

The final ensemble machine learning model was selected based on the ROC curve. Then future GPMs for the years 2025, 2030, 2035, and 2040 the best-fit model was simply multiplied by factors related to climate change in the raster calculator to develop the future groundwater potential models. The maps were reclassified using natural break techniques into five groups, representing regions with very high, high, moderate, low, and very low groundwater potential.

## Results

### Description of the parameters

**Figure 2:**
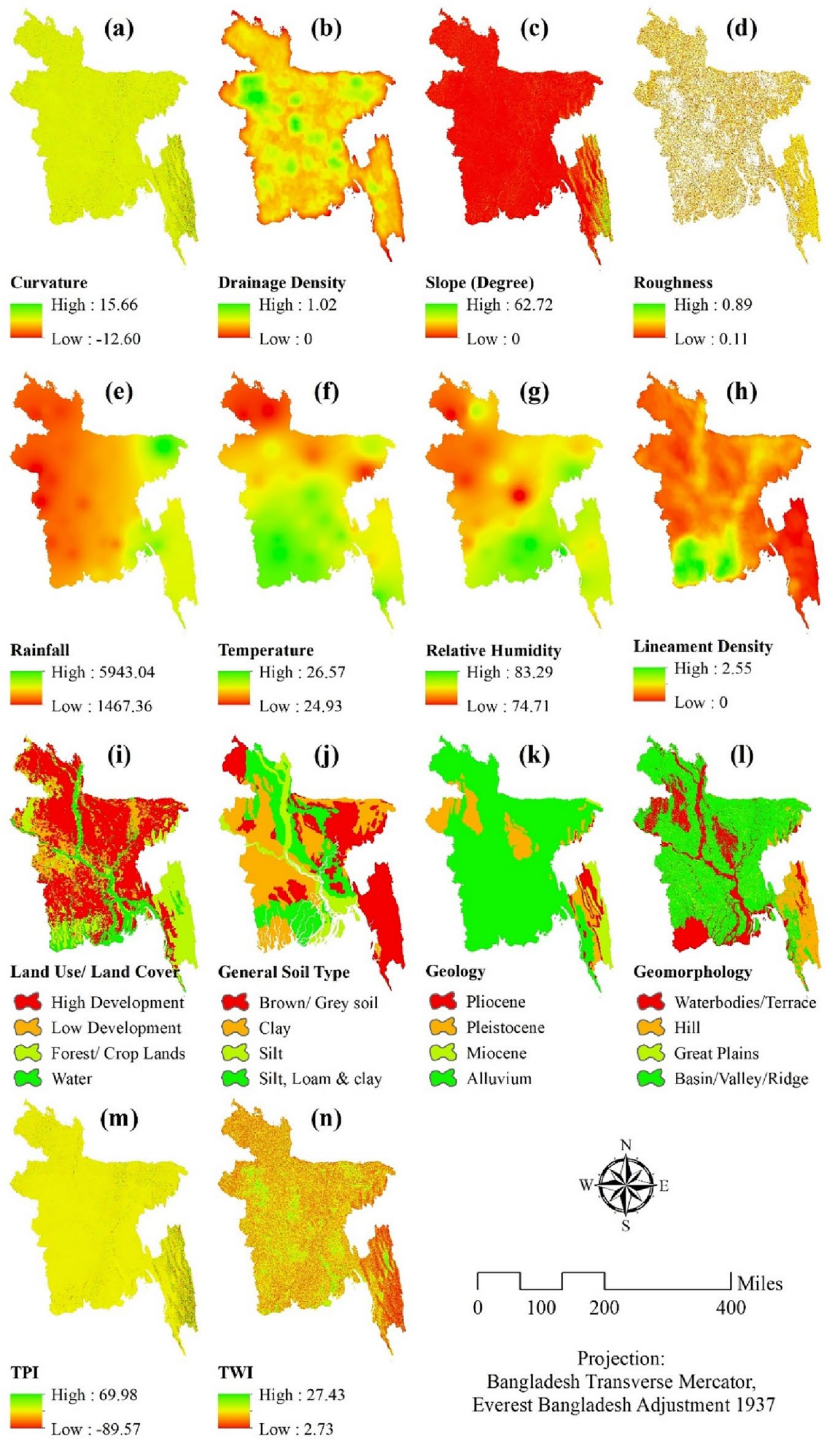
Spatial distribution of (**a**) curvature, (**b**) drainage density, (**c**) slope, (**d**) roughness, (**e**) rainfall, (**f**) temperature, (**g**) relative humidity, (**h**) lineament density, (**i**) land use and land cover, (**j**) general soil types, (**k**) geology, (**l**) geomorphology, (**m**) topographic position index (TPI), (**n**) Topographic Wetness Index (TWI); prepared by the authors using ArcGIS software version 10.5, (https://www.esri.com/en-us/arcgis/products).

The degree of the surface profile, which can be either convex or concave upward, is determined by its curvature. In convex and concave upward profiles, groundwater tends to slow down and collect, respectively^79^. The curvature value ranges from 15.66 to -12.60. In most of the cases, the value is in the medium range, though the south-eastern part of the region contains a higher level of curvature (Fig. 2a).

The availability and pollution of groundwater are highly dependent on drainage density. Low infiltration due to high drainage density does not enhance the area’s capacity for groundwater. Low drainage density adds more to groundwater potential since it reflects high infiltration. The lithology of a drainage system determines its quality and serves as a key indicator of the percolation rate^80^. The country’s western portion has a somewhat greater drainage density, but the southern section of the area has a low density. It is between 1.02 and 0 (Fig. 2b).

The steepness of the ground surface is expressed by the slope, a significant topographical element. Because rainwater travels fast down a steep slope after rainfall, larger slopes generate less recharge. Because of this, it does not have enough time to linger and replenish the saturated zone^4^. While most of the land is flat, the maximum slope value in the area is 62.72 degrees, which is largely found in the south-eastern and eastern sections of the nation. Larger sloped regions don’t have enough time for rainwater to enter and replace the suffocation zone because water flows over the top of a steeper slope quickly, which reduces the amount of groundwater recharge (Fig. 2c).

The roughness index in the Digital Elevation Model (DEM) compares the elevation difference between adjacent raster cells to represent the undulation of the topographic surface^6^. According to Fig. 2d, the roughness value ranges from 0.89 to 0.11. The topography’s overall undulation is expressed by the roughness index. The degree of undulation increases with increasing roughness, and vice versa^4^.

The concentration of rainfall is much higher in the south-eastern and eastern parts of the country. Rainfall values range from 5943.04 to 1467.36 (Fig. 2e). Temperature and humidity are two determining factors for precipitation and are thus significant when assessing a certain area’s groundwater potential.

The amount of subsurface water may change in response to an increase in surface temperature^81^, whereas prolonged periods of drought caused by low humidity might lead to excessive groundwater extraction or a shortage of it. The temperature varies throughout the whole country. The northern part of the country experiences lower temperatures, while the southern part of the country experiences a comparatively high level of temperature. But the temperature of the country can be defined as temperate as it ranges from 26.57 to 24.93 °C (Fig. 2f). Relative humidity is comparatively higher in places close to the sea, which is the southern part of the country, and the value decreases as it moves away from the sea. It ranges from 89.23 to 74.21 (Fig. 2g).

Lineament density reveals the faulting and fracture zones that provide the ground surface its secondary porosity and permeability^80^. Near high-density lineament areas, there is significant potential for groundwater, and vice versa^79^. Lineament density is higher in the south-western part of the region; other than that, the value is low in other parts of the region. It ranges from 2.55 to 0 (Fig. 2h).

The amount and quality of groundwater can be estimated from the pattern of land use and cover in a given location. Studies have shown that when urbanization (rise in built-up areas), population growth, and agricultural practices increase in various geographic contexts, groundwater levels, quality, and recharge capacity drop with time^6,82^. (Fig. 2i) reveals that, with the exception of the Chittagong Hill Tracts region to the southeast, more than 50% of Bangladesh’s land area is subject to high intensity development, indicating that this enormous area has a lesser capacity for groundwater recharging by infiltration than other areas.

The amount of water that may enter underground formations depends on the kind of soil, which also affects groundwater recharge^2^. Soil texture and hydraulic characteristics are the two main factors that are looked at when determining the rate of infiltration. In Bangladesh, the general soil type may be divided into four categories: brown, clay, slit, and mixed varieties. The region’s southern and eastern regions have brown-gray dirt. In the eastern portion of the area, there is soil that resembles clay. The southern portion of the area, which is primarily referred to as the delta region, is where mixed-type soil is typically found (Fig. 2j).

Groundwater recharge and prevalence are mostly determined by an area’s geological makeup^83^. There are four different types of geology in the area, including Pliocene, Pliestocene, Miocene, and Alluvium. The majority of the area is covered with alluvium, but the western and central portions of the area are dominated by pliesocene. In the southeast corner of the area, there are four different types of geology present (Fig. 2k).

The study area showcases a wide array of geomorphological characteristics, which can be classified into four primary categories: water bodies and terraces, hills, great plains, and a combination of basins, valleys, and ridges (Fig. 2l). Waterbodies are particularly apparent across the central region of the study area, offering crucial resources and habitats. In addition, the mangrove areas in the southwestern region are primarily characterized by the presence of water bodies. The research area’s southeastern region exhibits topography that is predominantly hilly. These areas provide a distinct difference from the flat plains and water-covered regions, contributing to the topographical variety of the region. The areas with basins, valleys, and ridges, occupy the majority of the study region and contribute to a complex structure of geographical characteristics that exhibit significant influence over the landscape.

An method called TPI is frequently used to calculate topographic slope locations and automatically classify landforms^84^. The TPI value ranges from 69.98 to − 89.57 (Fig. 2m), and the value is moderate and comparatively higher in the south-eastern part of the region. TWI is often used in the calculation of the effects that topography has on hydrological processes and in the representation of the potential for groundwater penetration^85^. TWI ranges from 27.43 to 2.73 (Fig. 2n).

### Groundwater potentiality modeling

**Figure 3:**
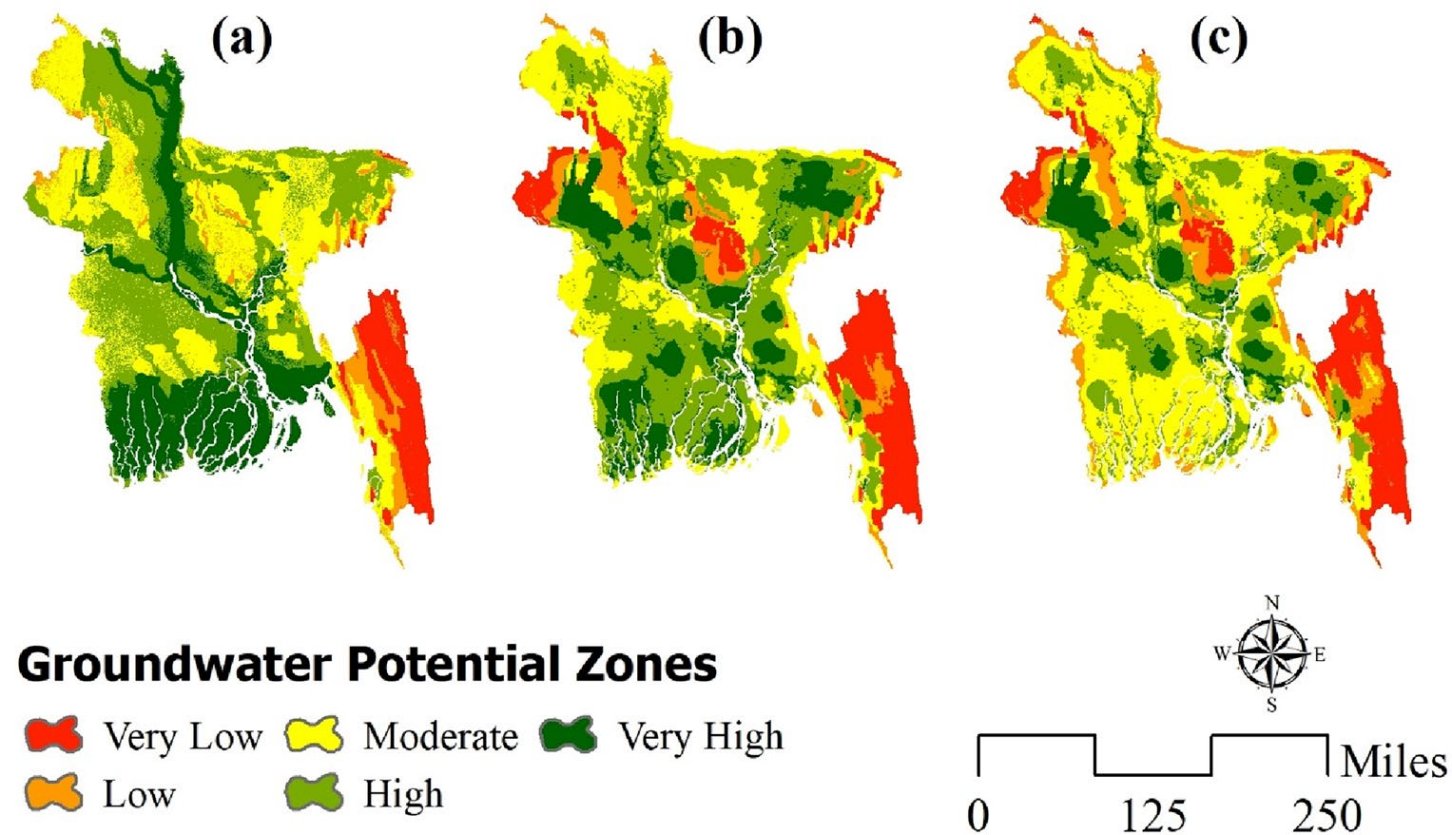
Model result of (**a**) ANN; (**b**) LR; (**c**) LMT; prepared by the authors using ArcGIS software version 10.5, (https://www.esri.com/en-us/arcgis/products).

Figure 3 illustrates the spatial distribution of groundwater potential zones as determined by three cutting-edge machine learning techniques. Jenks’ natural breaks classifier was used to categorize groundwater potential maps into five classifications (very low, low, moderate, high, and very high).

**Figure 4:**
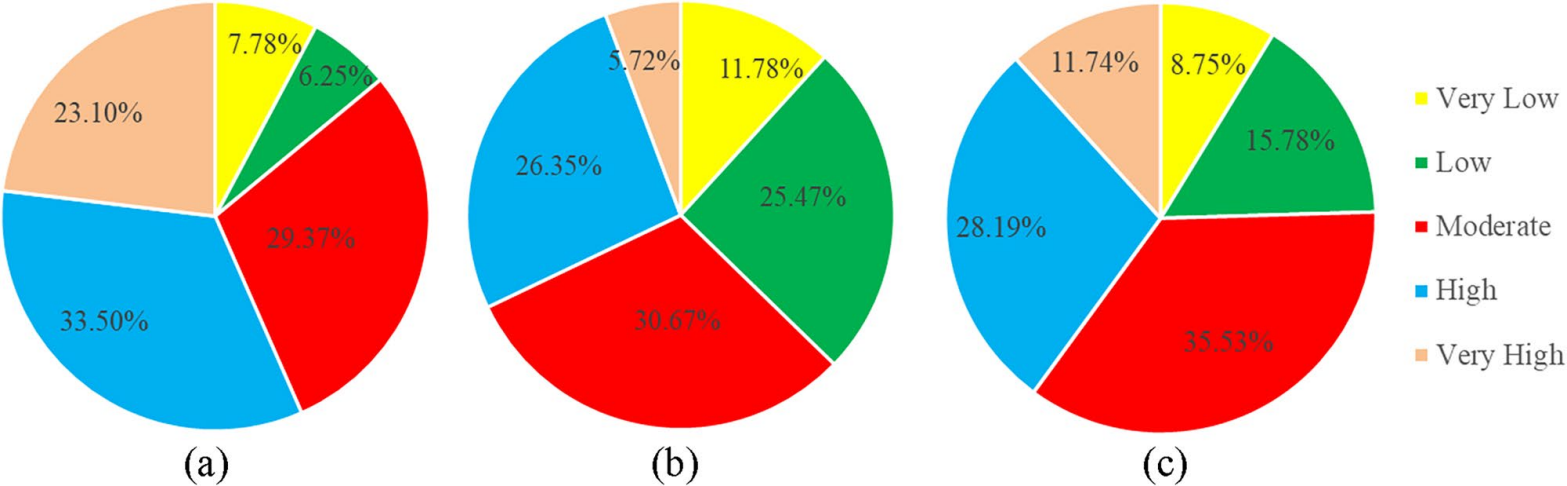
Area in percentage for (**a**) ANN; (**b**) LR; (**c**) LMT.

According to the Artificial Neural Network (ANN) model, regions with a high potential for groundwater were identified in 23.10% and 33.50% of the areas, respectively. Additionally, moderate (29.57%), low (6.25%), and very low (7.78%) potential zones for groundwater were also observed (as shown in Fig. 4). Based on the spatial distribution of groundwater potential zones illustrated in Fig. 3a, it can be observed that the Brahmaputra, Jamuna, Padma, and Meghna rivers, along with other major rivers traversing the entire country, are predominantly situated adjacent to areas classified as very high and high zones in close proximity to the Bay of Bengal. This pertains specifically to the southern region of Bangladesh. The high and very high groundwater potential zones were significantly impacted by prominent bodies of water. The regions of north-east, central, and north-west Bangladesh have been found to exhibit predominantly moderate potential for groundwater. The regions of Rangamati, Khagrachari, and Bandarban, characterized by their mountainous terrain, have been found to exhibit low potential for groundwater due to the lack of significant river systems. Moderate groundwater zones are present in various areas of Chittagong and Cox’s Bazaar district due to the influence of the Bay of Bengal.

As per the LR model, it has been observed that certain areas exhibit a significantly elevated groundwater potential, with 5.72% and 26.35% of the landmass falling under the categories of very high and high potential zones, respectively. The analysis of the geographical distribution demonstrated that, akin to the ANN model, regions classified as having extremely high and high values were situated in proximity to major river systems. The lower part of the south-west coastal district, comprising Khulna, Satkhira, and Bagerhat, is classified as high or very high potential zones, while the central coastal district, consisting of Barguna, Patuakhali, and Barishal, is categorized as moderate zones. The northeastern corner of the region exhibits significant potential. Similar to the ANN model, hilly districts were situated in regions characterized by significantly limited and deficient groundwater potential. Nonetheless, the model classified certain areas situated in the northwestern region of the province, namely Nawabganj, Rajsahi, and Naogoan, as zones with exceedingly low potential.

The LMT model was able to classify the areas into different groundwater potential zones. Specifically, 11.74% and 28.19% of the areas were classified as having very high and high groundwater potential regions, respectively. The remaining areas were classified as having moderate (28.19%), low (15.78%), as well as very low (8.75%) groundwater potential zones. The present model indicates that the coastal regions of the nation were situated in the moderate zone, a deviation from the preceding models. As per the LMT model’s prediction, the northwestern region exhibited a significantly low potential zone. The southern-eastern hill districts have been identified as having low to very low potential, which is consistent with the potential zones of ANN and LR. The study revealed the presence of significantly elevated zones in the vicinities surrounding the primary rivers and in the north-eastern regions, characterized by haors.

### Validation of the models

**Figure 5:**
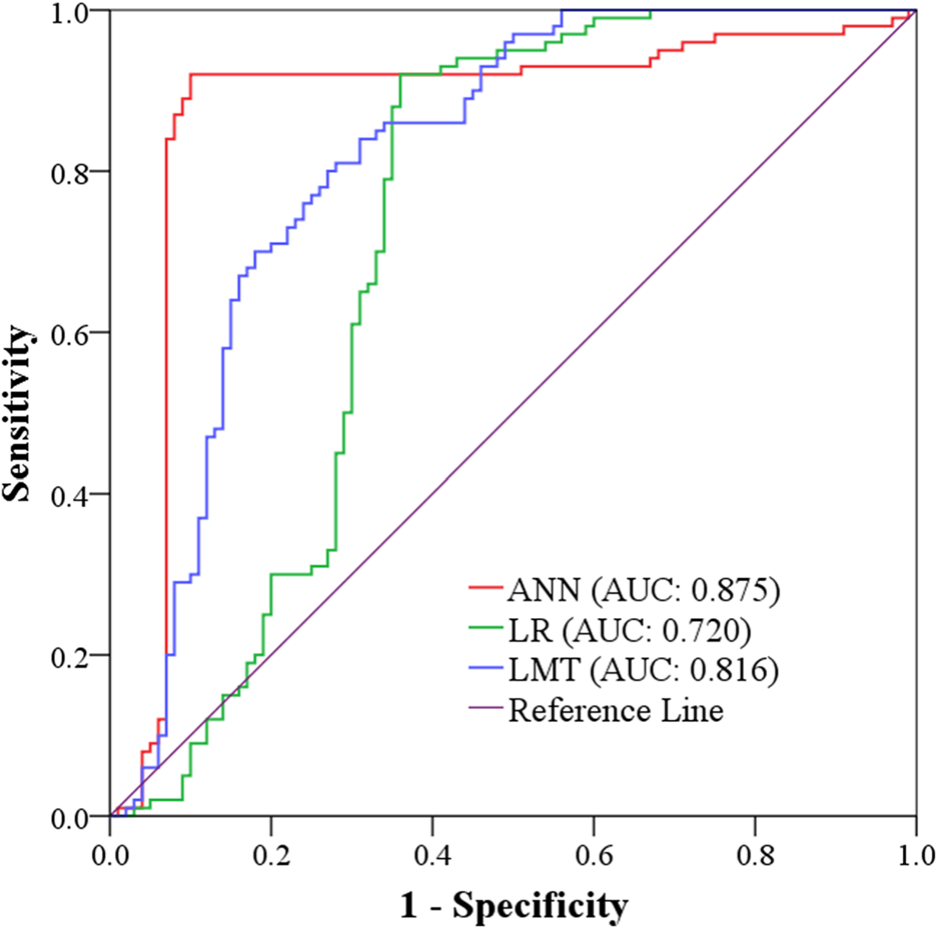
ROC curve of ANN, LR, and LMT.

The created groundwater potential maps were validated using the area under curve (AUC) of receiver operating characteristic (ROC). The AUC values for the ROC curves are reported for three classification models: artificial neural network (ANN), logistic regression (LR), and decision tree (LMT). The corresponding AUC value for ANN is 0.875, for LR is 0.720, and for LMT is 0.816 (Fig. 5). According to both ROC curves, ANN is said to be more accurate and precise than other models. But the ROC value is higher than 0.7 in all three models, which states that the results given by all three models can be considered acceptable.

### Future climate change scenarios

**Figure 6:**
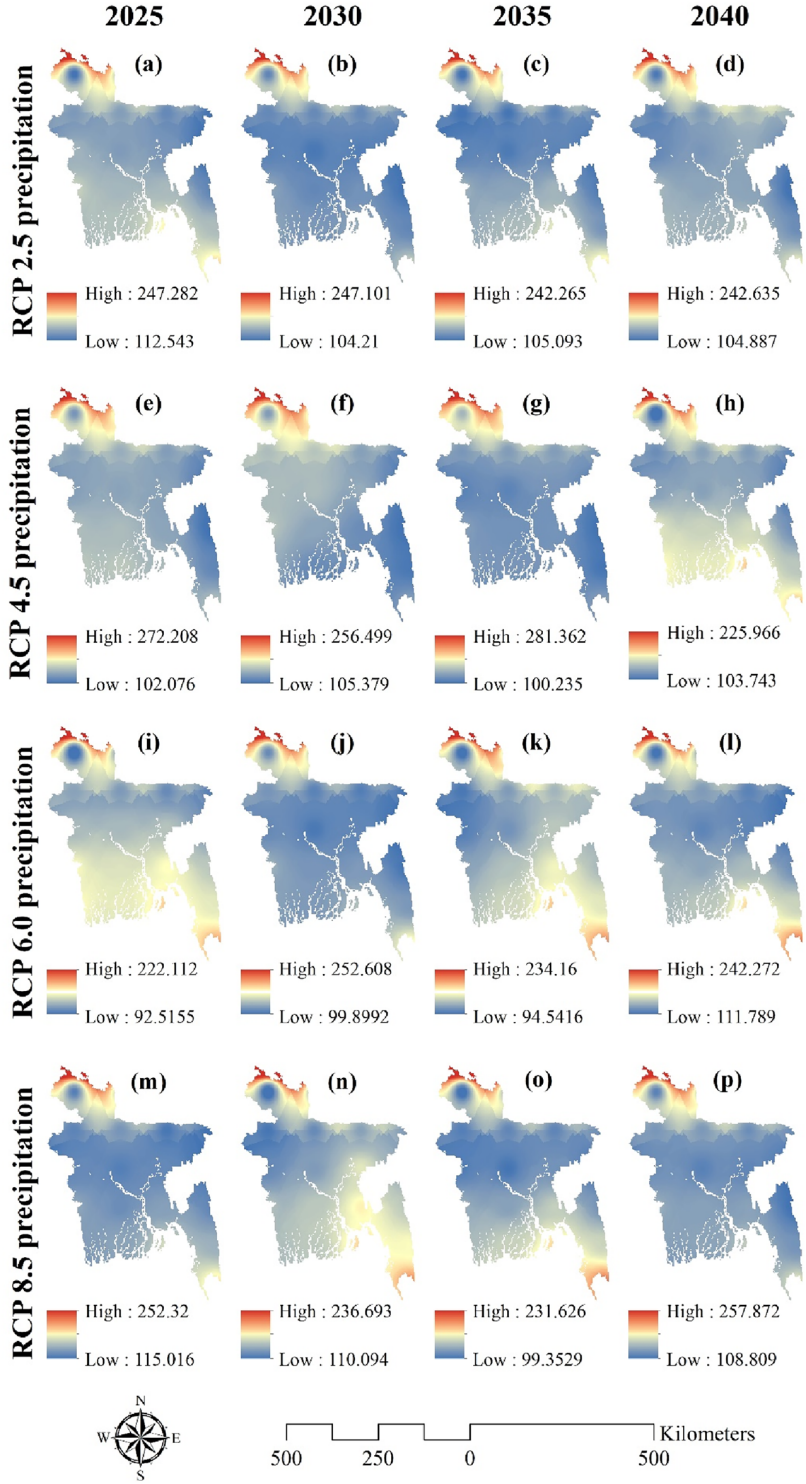
Spatial distribution of future climate change variables for 2025, 2030, 2035, and 2040, such as (**a**–**d**) RCP2.5 precipitation (mm); (**e**–**h**) RCP4.5 precipitation (mm); (**i**–**l**) RCP6 precipitation (mm) and (**m**–**p**) RCP8.5 precipitation (mm); prepared by the authors using ArcGIS software version 10.5, (https://www.esri.com/en-us/arcgis/products).

Precipitation and groundwater potential zones were estimated using the ANN model for four different climate change scenarios (RCP2.5, RCP4.5, RCP6, and RCP8.5). Three scenarios are projected: RCP2.5 (the most optimistic), RCP8.5 (the most pessimistic), and RCP4.5 (the stability scenario)^85^. This study forecasted the future for four different years, including 2025, 2030, 2035, and 2040. Figure 6 depicts precipitation predictions for four climate change scenarios (RCP2.5, RCP4.5, RCP6, and RCP8.5) across four years. The highest value of precipitation in 2025 for the RCP 2.5 scenario was 247.82, which reduced to 242.635 in 2.40, while the lowest value likewise decreased from 112.543 to 104.887 in 2.40. Greater values were seen in the northern, north eastern, and south eastern zones. Figure 6e–h display the RCP 4.5 scenario.

The maximum precipitation value was 272.208 in 2025, which grew to 281.362 in 2035 but decreased to 225.966 in 2040. Figure 6i–l depict the RCP 6 scenario. Precipitation peaked at 111.789 in 2040 after dropping to 92.515 in 2025. Contrarily, the maximum figure was 222.112 in 2025, rose to 252.608 in 2030, and then fell to 242.272 in 2040. In 2025, it was observed that the region’s northern and southern sections had higher precipitation values than other regions, but by 2040, this trend had reversed, notably in the region’s southern parts. Figure 6m–p depict the RCP 8 scenario. In 2025, the greatest value was 252.32, and in 2040, it rose to 257.872. But it did slightly decline in 2030 and 2035. Precipitation values were much higher region-wise in 2030, but they decreased by 2040.

### Future groundwater potential scenarios

**Figure 7:**
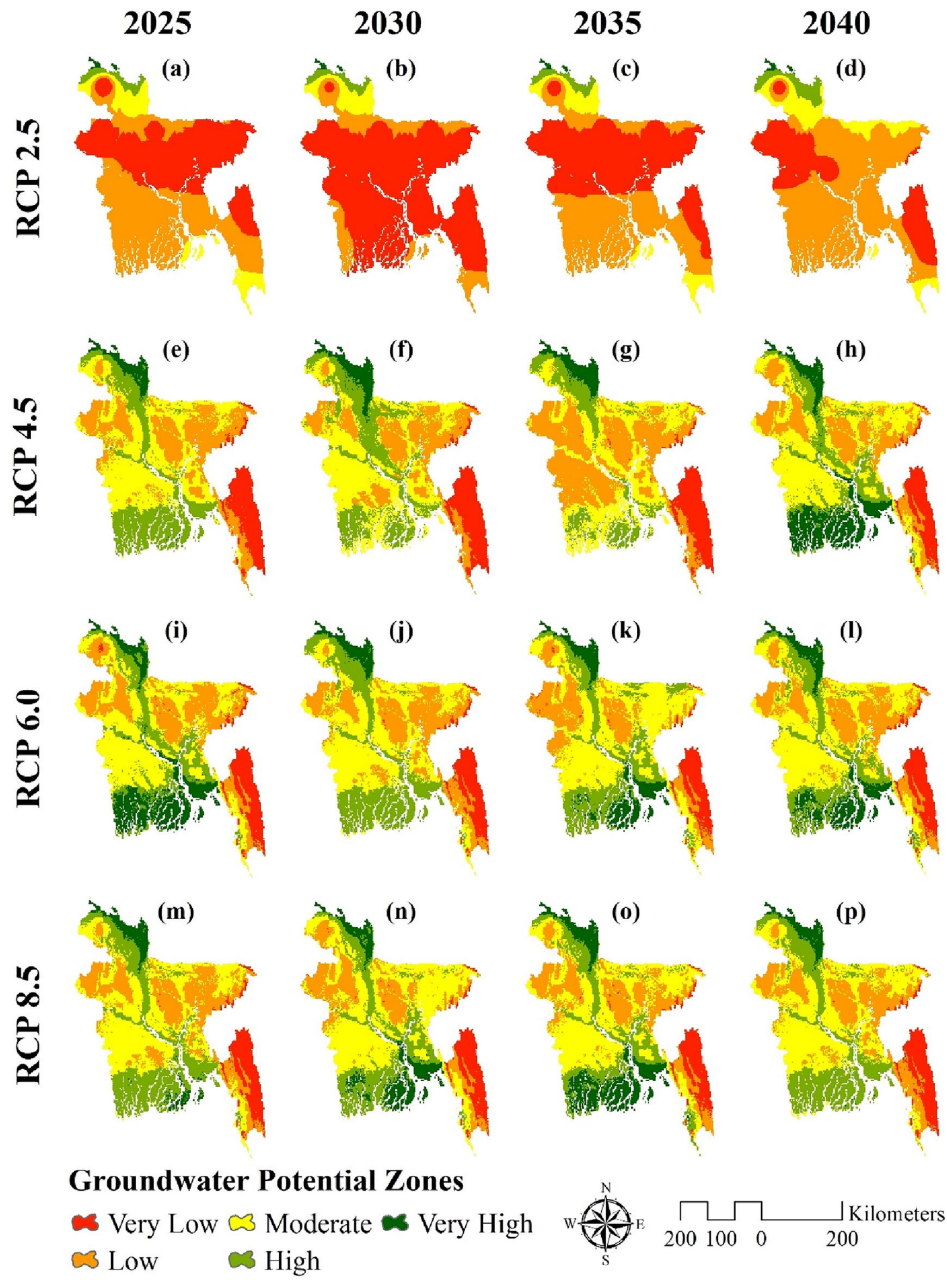
Spatial distribution of future groundwater potential for 2025, 2030, 2035, and 2040, such as (**a**–**d**) RCP2.5 precipitation (mm); (**e**–**h**) RCP4.5 precipitation (mm); (**i**–**l** RCP6 precipitation (mm) and (**m**–**p**) RCP8.5 precipitation (mm); prepared by the authors using ArcGIS software version 10.5, (https://www.esri.com/en-us/arcgis/products).

Figure 7 displays the forecasts of groundwater potential zones for four climate change scenarios (RCP2.5, RCP4.5, RCP6, and RCP8.5) over a fifteen-year period. Groundwater potential is predicted to be low in the region’s center, east, and west under RCP 2.5 by the year 2025. In 2030, the situation worsened since, with the exception of the northern half of the region, practically the entire country is comprised of very low potential zones. However, the scenario changed between the years 2035 and 2040. According to Fig. 7e–h, the groundwater potential zone improved from 2025 to 2040. Coastal regions with high potential zones were ranked up to very high potential zones. However, between 2030 and 2035, the majority of the region’s moderate potential zone shifted to the low potential zone. The RCP 6 scenario of groundwater potential zone is depicted in Fig. 7i–l. Coastal regions that were in the very high potential zone in 2025 were shifted to the high potential zone in 2030. In 2035, a large area in the west switched from a moderate to a low potential zone. The north-eastern region’s moderate zones have transitioned from moderate to low potential zones. The RCP 8 scenario is depicted in Fig. 7m–p. The situation was substantially better in 2030 and 2035 of the RCP8 scenario than in 2025 and 2040. Almost the whole coastal region that was a high potential zone in 2035 became a very high potential zone. But at this time some of the zones that were moderate in 2030 transformed into low potential zone.

## Discussion

Groundwater levels have decreased in many places across the world, particularly in semi-arid regions, as a result of overuse of groundwater supplies for agriculture, businesses, and municipal purposes in recent decades^86^. Bangladesh is endowed with a diverse range of water sources owing to its riverine topography and tropical climate. Nevertheless, the over-extraction of groundwater, coupled with the impact of climate change, has resulted in water scarcity during dry spells. Therefore, it is imperative to evaluate the groundwater zones’ capacity to address this issue. It’s crucial to identify, categorize, and evaluate the traits and elements that affect groundwater^78^. To identify Bangladesh’s groundwater potential zone, several research have been conducted. This study employed three distinct machine learning algorithms, namely artificial neural network (ANN), logistic regression (LR), and logistic model tree (LMT), to identify potential zones for groundwater. Fourteen factors, including climate, drainage density, slope, roughness, rainfall, temperature, relative humidity, lineament density, land use/land cover, general soil type, geology, geomorphology, TPI, and TWI, were utilized in the analysis. The Artificial Neural Network (ANN) model identified regions with varying degrees of groundwater potential. Specifically, the model detected zones with very high groundwater potential in 23.10% and 33.50% of the regions, respectively. These were followed by zones with moderate (29.57%), low (6.25%), and very low (7.78%) groundwater potential, as presented in Fig. 4. The LR model has identified zones with high and very high potential for groundwater in 26.35% and 5.72% of the areas, respectively. The LMT model was able to detect areas with significant groundwater potential. Specifically, the model identified zones with high and very high groundwater potential in 11.74% and 28.19% of the areas, respectively. Additionally, moderate (28.96%), low (15.78%), and very low (8.75%) groundwater potential zones were also identified. All three models show a lot of resemblance; for example, the south-eastern region of Bangladesh consists of very low potential zones, while the areas adjacent to major rivers consist of very high potential zones, which are similar to those in previous studies as well^6,87^. But the ANN model shows that the coastal region in the south-western part consists of a very high potential zone, whereas LR shows it is a mix of high and very high potential zones, and finally LMT categorizes it as a moderate potential zone. A previous study done by^6^ using AHP classified it as a mix of the very high and high potential zones. Another dissimilarity between ANN and the other two models is that the western part of the region consists of very low potential zones in LR and LMT. But in ANN, it is classified as a moderate potential zone. After detecting potential zones, a comparison between three models is done to identify which model gives the best result, using the ROC curve. The ROC curve is frequently used for the accuracy assessment^6,88,89^. The ROC curve shows that (empirical and binominal) is 0.884 and 0.877 for ANN, 0.858 and 0.65 for LR, and 0.814 and 0.71 for LMT. So it can be said that ANN shows better accuracy than the other two models. Prior research done by^90–92^ also said ANN provided better results for groundwater potential zones. The ANN model excelled in capturing intricate relationships and patterns within the dataset. Its ability to handle non-linearity and interactions among variables contributed to its superior performance. However, the ANN model’s "black-box" nature makes it challenging to interpret the specific features driving predictions^93,94^. Additionally, training an ANN requires more computational resources compared to LR and LMT. LR and LMT, while not outperforming ANN, demonstrated reasonable accuracy. LR is interpretable, allowing for a straightforward understanding of feature contributions. LMT combines decision tree interpretability with logistic regression, offering a balance between complexity and interpretability. LR may struggle with capturing complex relationships, while LMT might be sensitive to overfitting^76^.

Sensitive issues have emerged, especially in areas where groundwater sources account for the bulk of water supplies, and changes in the groundwater hydrological system have been acknowledged as one of the repercussions of climate change in a variety of locations^95,96^. The component most susceptible to upcoming climate changes has been determined to be precipitation patterns^97^. ANN produced a map that was then used for four different RCP scenarios (RCP 2.6, RCP 4.5, RCP 6, and RCP 8.5) to see how the precipitation and groundwater potential zones would react to different climate change scenarios in 2025, 2030, 2035, and 2040^95^. The IPCC’s Representative Concentration Pathway (RCP) is a greenhouse gas concentration trajectory. Four climate modeling and research approaches were used in the 2014 IPCC Fifth Assessment Report. From Fig. 4, it can be said that RCP4.5 and RCP6 show completely reversed results. In the RCP4.5 scenario, precipitation tends to increase, whereas in the RCP6 scenario, precipitation tends to decrease over the years. In RCP2.5 and RCP8, precipitation remains in a standard situation. Figure 5 depicts the groundwater potential zones in four different scenarios. It can be said that the RCP2.5 scenario is the worst among the rest of the scenarios, especially in 2030, when most of the region would be converted into a very low potential zone. The situation would somehow get better in 2035 and 2040, but still, it consists of a lot of low and very low potential zones. Much improvement can be seen in the RCP4.5 scenario. But the RCP6 and RCP8 scenarios show continuous deterioration, which is consistent with the findings of^33,78,98^. The study has shed light on the potential impact of climate change on groundwater potential zones in Bangladesh. The observed trends under different Representative Concentration Pathway (RCP) scenarios highlight the sensitivity of groundwater availability to changes in precipitation patterns. The results indicate that certain scenarios, such as RCP4.5, may show an improvement in groundwater potential, while others, like RCP6 and RCP8, suggest continuous deterioration. It’s crucial to emphasize that these findings have significant implications for water resource management, especially in regions heavily reliant on groundwater.

This is the first study of its sort to compare the accuracy of three different machine learning models, forecast possible zones for groundwater use in the coming years using the model that performed the best, and anticipate how groundwater and precipitation will respond to four RCP scenarios in the upcoming years (2025, 2030, 2035, and 2040). Various machine learning models could have various algorithms, parameters, and training sets, so this study would help the policy makers decide which model is best fit to detect Bangladesh’s groundwater potential zones as well as globally. The nation is susceptible to the effects of climate change, such as increased temperatures and altered precipitation patterns. These modifications may affect the availability and rates of groundwater recharge, thus resulting in groundwater scarcity in some areas. Water managers can anticipate the effects of climate change on groundwater supplies and make plans for sustainable management of water resources based on this study. This study has certain drawbacks as well because it only evaluated the three most frequently used machine learning models; nevertheless, there are more models that can be compared, and there hasn’t been a physical survey. While the ROC curve analysis indicates the superior performance of ANN, it’s essential to acknowledge uncertainties and variations in model predictions. Conducting a sensitivity analysis could enhance the robustness of the models by exploring how changes in input variables influence predictions. The identified groundwater potential zones can serve as valuable inputs for water resource management in Bangladesh. Decision-makers can use this information to implement sustainable water resource utilization strategies, especially in regions prone to water scarcity. Future research should improve climate models and precipitation projections to overcome uncertainties from Representative Concentration Pathway (RCP) scenarios. Hydrological and climatic models must be integrated to comprehend precipitation, surface water, and groundwater interactions. Adding climatic factors like evapotranspiration and soil moisture might enhance groundwater potential forecasts. Model validation and refining will use empirical data from long-term monitoring programs that include local knowledge and measurements. The socio-economic effects of groundwater potential zone changes on agriculture, livelihoods, and community resilience must be examined. Dynamic modelling frameworks that allow for adaptation strategies and worldwide comparative studies can help comprehend global groundwater dynamics under climate change. These research initiatives resolve uncertainties, increase model robustness, and give practical insights for climate-resilient water resource management.

## Conclusion

For developing machine-learning-based groundwater potential zoning, this study considered fourteen different factors. This was found in all three models: southern hill tracts are facing a lot of groundwater stress and potential zones are higher adjacent to major rivers and harbor areas, though coastal region shows different results in all three models. A comparison between three different ML models using the ROC curve found that the ANN gave a better result than the LR and LMT. The results given by LR and LMT are also useful, as they give an accuracy of over 80%. A model’s capacity to distinguish groundwater potential zones is shown by the ROC curve. At various thresholds, it shows true positive rate (sensitivity) vs false positive rate (1-specificity). Our study found that models with a bigger area under the ROC curve (AUC) better discriminate groundwater potentialities. ANN’s greater AUC reflects improved sensitivity and specificity, resulting in more accurate groundwater potential zone forecasts than LR and LMT. Precipitation in the RCP6 scenario is considered to be worse, and RCP4.5 is considered to be the best in terms of high precipitation values across the country. RCP2.5 shows the worst result in the case of the groundwater potential zone, as most of the region consists of low and very low potential zones. RCP4.5, on the other hand, provides the best result for the groundwater potential zone. A major reason can be said to be the high precipitation ratio across the country in this scenario. Understanding these scenarios aids policymakers in crafting effective long-term plans aligned with climate change projections. The study’s findings can assist policymakers in Bangladesh in creating efficient groundwater management plans, maximizing existing infrastructure for groundwater extraction, selecting the best locations for groundwater monitoring and data collection, ensuring the long-term sustainability of groundwater resources, and creating efficient water management plans that encourage efficient management techniques and take into account potential climate change effects on groundwater resources. We suggest that, in future investigations, the comparative analysis be expanded to incorporate Support Vector Machines, Random Forest, and Decision Trees. Furthermore, the inclusion of novel variables such as alterations in land use and aquifer properties within RCP scenarios can augment the precision of predictions, thereby fostering a more holistic comprehension of groundwater dynamics and providing valuable insights for the development of sustainable approaches to water resource management.

## Data Availability

The datasets used in the study are available from the corresponding author upon reasonable request.
